# Myocardial creep-induced misalignment artifacts in PET/MR myocardial perfusion imaging

**DOI:** 10.1007/s00259-020-04956-y

**Published:** 2020-07-18

**Authors:** Elia von Felten, Georgios Benetos, Dimitri Patriki, Dominik C. Benz, Georgios P. Rampidis, Andreas A. Giannopoulos, Adam Bakula, Christoph Gräni, Aju P. Pazhenkottil, Catherine Gebhard, Tobias A. Fuchs, Philipp A. Kaufmann, Ronny R. Buechel

**Affiliations:** Department of Nuclear Medicine, Cardiac Imaging, University Hospital Zurich, University of Zurich, Ramistrasse 100, CH-8091 Zurich, Switzerland

**Keywords:** PET/MR, Myocardial perfusion imaging, Attenuation correction, Artifact

## Abstract

**Purpose:**

Misalignment between positron emission tomography (PET) datasets and attenuation correction (AC) maps is a potential source of artifacts in myocardial perfusion imaging (MPI). We assessed the impact of adenosine on the alignment of AC maps derived from magnetic resonance (MR) and PET datasets during MPI on a hybrid PET/MR scanner.

**Methods:**

Twenty-eight volunteers underwent adenosine stress and rest 13N-ammonia MPI on a PET/MR. We acquired Dixon sequences for the creation of MRAC maps. After reconstruction of the original non-shifted PET images, we examined MRAC and PET datasets for cardiac spatial misalignment and, if necessary, reconstructed a second set of shifted PET images after manually adjusting co-registration. Summed rest, stress, and difference scores (SRS, SSS, and SDS) were compared between shifted and non-shifted PET images. Additionally, we measured the amount of cranial movement of the heart (i.e., myocardial creep) after termination of adenosine infusion.

**Results:**

Realignment was necessary for 25 (89.3%) stress and 12 (42.9%) rest PET datasets. Median SRS, SSS, and SDS of the non-shifted images were 6 (IQR = 4–7), 12 (IQR = 7–18), and 8 (IQR = 2–11), respectively, and of the shifted images 2 (IQR = 1–6), 4 (IQR = 7–18), and 1 (IQR = 0–2), respectively. All three scores were significantly higher in non-shifted versus shifted images (all *p* < 0.05). The difference in SDS correlated moderately but significantly with the amount of myocardial creep (*r* = 0.541, *p* = 0.005).

**Conclusion:**

Misalignment of MRAC and PET datasets commonly occurs during adenosine stress MPI on a hybrid PET/MR device, potentially leading to an increase in false-positive findings. Our results suggest that myocardial creep may substantially account for this and prompt for a careful review and correction of PET/MRAC data.

## Introduction

When combined and fully integrated positron emission tomography (PET)/magnetic resonance (MR) devices have become commercially available, the calculation of accurate attenuation correction (AC) maps was found to be a significant challenge for PET/MR imaging. While AC based on dedicated computed tomography (CT) transmission data is firmly established for PET/CT and has been shown to be robust [1], creating AC maps with MR data from a PET/MR imposes a series of challenges such as the lack of MR signal for the patient’s table and MR coils, field inhomogeneities, limited field of view, and finally the difficulty in obtaining accurate lung and bone segmentation. One potential solution to the latter consists of water-weighted and fat-weighted datasets derived from Dixon MR sequences [2, 3]. The fact that MR sequences for the creation of AC maps are acquired over several breathing cycles may theoretically even constitute an advantage over CT-derived AC maps as the maps should correlate better with the commonly non-respiratory-gated PET data. AC derived from Dixon sequences has been validated for whole-body as well as for cardiac PET/MR [4, 5]. These studies have focused on 18F-fluorodeoxyglucose (FDG) PET and rest PET myocardial perfusion imaging (MPI). For comprehensive MPI in a setting of known or suspected coronary artery disease (CAD), however, a stress acquisition is mandatory and is most commonly performed using vasodilator infusion. It is well known that vasodilators may cause a change in respiratory levels with the diaphragm, and subsequently the heart shifting to a more caudal position during stress followed by a gradual cranial movement once the vasodilator stimulus is terminated. This phenomenon, termed myocardial creep, has been described recently for PET MPI [6–8] but was initially observed in single-photon emission tomography (SPECT) MPI after physical stress [9]. While it has been shown that manual frame-by-frame correction of the left-ventricular myocardial contours is mandatory for quantitative MPI to compensate for the motion of the heart during myocardial creep [8], the effect of such motion on accurate alignment of AC and PET datasets is less well investigated, particularly for novel combined PET/MR scanners. As with PET/CT scanners, PET/MR devices offer inherent spatial co-registration of AC and PET datasets. Because the patient is literally tied to the scanner by the body coils, gross patient movement during the acquisition is unlikely, and misalignment of the co-registered PET and AC datasets is not necessarily expected. However, respiratory changes have been described as a cause of cardiac misalignment between CTAC maps and PET datasets [10, 11]. Although the protocols and the techniques of PET MPI using a PET/CT scanner differ fundamentally from PET/MR, myocardial creep may potentially trigger cardiac misalignment in a hybrid PET/MR scanner as well.

In this study, we aimed to assess the frequency of cardiac misalignment in PET/MR MPI, its impact on image quality, and potential mechanisms causing the related artifacts.

## Methods

### Study population

Thirty volunteers who underwent PET/MR MPI were assessed. All patients were recruited as part of a study evaluating potential long-term effects of myocarditis on the left-ventricular myocardium. Two patients were excluded because of incomplete data acquisition. Hence, the final study population consisted of 28 patients. All patients had documented myocarditis in the past (> 6 months). All patients were at least 18 years of age and did not have any contraindications against MR imaging (e.g., no implanted cardiac devices, claustrophobia, known allergy against gadolinium-based contrast agents, or severely impaired renal function), adenosine (e.g., no asthma or higher-grade atrioventricular block), or PET (e.g., no pregnancy or breastfeeding). This study was approved by the local ethics committee (BASEC-Nr. 2018-00170). All patients provided written informed consent.

### Data acquisition

**Fig. 1:**
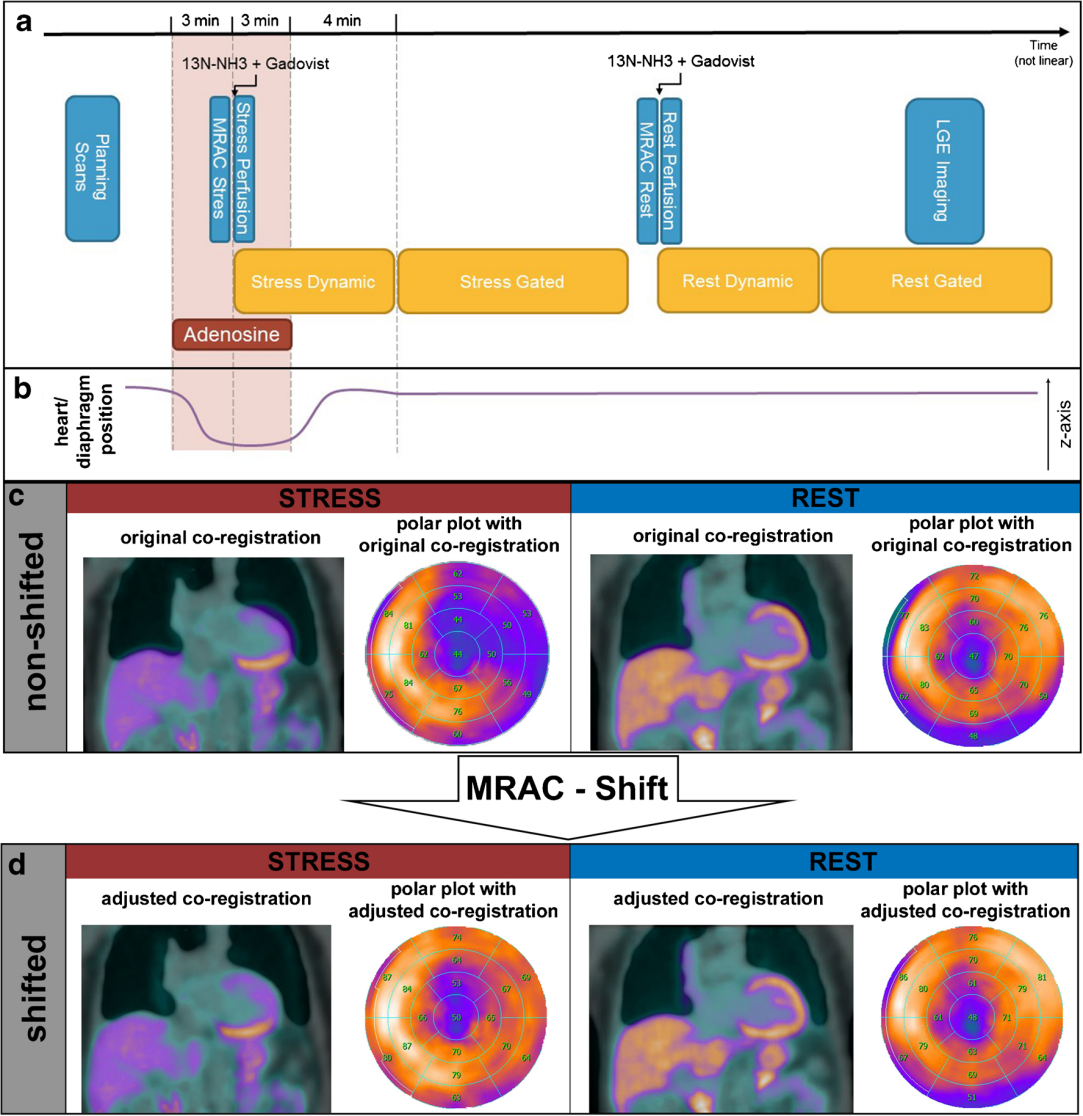
Myocardial creep-induced MRAC misalignment artifact (MCMA)—mechanism of origin and correction. **a** PET/MR MPI protocol. MR imaging is depicted in blue boxes and PET imaging in yellow boxes. **b** Heart position along the *z*-axis during acquisition. During adenosine stimulation, the heart moves away from its initial position, and after termination of the adenosine infusion, it slowly creeps back (e.g., myocardial creep). **c** Cardiac misalignment between MRAC and PET images based on the original co-registration with corresponding polar plots for stress and rest. The artifact can be localized on the anterolateral wall. **d** After MRAC shifting. Proper alignment of the cardiac contours in MRAC and PET images based on the adjusted co-registration with corresponding polar plots for stress and rest

All patients were instructed to fast for at least 6 h and refrain from any caffeine intake for at least 12 h before undergoing a combined PET/MR MPI protocol (Fig. 1A) on a hybrid device incorporating a 3-Tesla MR and a PET scanner (SIGNA PET/MR, GE Healthcare, Waukesha, WI, USA).

MR acquisition for attenuation correction (MRAC) was performed immediately before the start of PET data acquisition. No cardiac or respiratory gating was applied, as it was not available for MPI. The in-phase, out-of-phase, and the derived DIXON-based water- and fat-only image datasets used for MRAC were generated by the automatically performed multi-station, whole-body, three-dimensional, dual-echo radio-frequency spoiled gradient recalled echo sequence. An air, lung, and continuous fat/water model was applied to generate a four-compartment attenuation map for PET isotopes. A truncation completion algorithm was used as previously described [12, 13]. PET perfusion imaging was initiated after 3 min into adenosine stress (140 μg/kg/min). PET data were acquired in list mode and reconstructed as a static, dynamic (7 min divided into 21 frames 9 × 10 s, 6 × 15 s, 3 × 20 s, 2 × 30 s, and 1 × 120 s), and an ECG-gated dataset (10 min) using TOF reconstruction with VUE Point FX (2 iterations and 16 subsets) and a 5-mm Hanning filter after injection of 259 ± 22 MBq 13N-NH3. Following stress data acquisition, PET rest perfusion acquisitions were performed similarly but using 473 ± 71 MBq of 13N-NH3.

### MRAC shift

First, static PET images based on the original co-registration were reconstructed on the PET/MR console without any manual corrections (non-shifted images). In a second step, and after transferring the PET and MR data to a dedicated workstation (Advantage Workstation 4.7, GE Healthcare), the static PET images were carefully examined for cardiac misalignment with the MRAC maps (Fig. 1C), by two cardiac imaging physicians with expertise in cardiac PET and MR imaging by consensus, using commercially available software (General Registration Tool, GE Healthcare). Cardiac misalignment was defined as a visually perceivable misalignment between the left-ventricular contours of the MRAC maps and the PET datasets. If cardiac misalignment was present, a manual rigid MRAC shift was performed along the *x*-, *y*-, and *z*-axis to adjust the spatial co-registration. Cardiac misalignment was quantified as the summed correction distance along all three axes in millimeters. Finally, the second set of static images (shifted images) was reconstructed (Fig. 1D).

### PET analysis

Shifted and non-shifted images were analyzed using a commercially available software tool (Cedars-Sinai Cardiac Suite, version 2017.2, Cedars-Sinai Medical Center Artificial Intelligence in Medicine Program, Los Angeles, CA, USA). Based on a 17-segment model, two experienced readers determined stress and rest scores for each segment by scoring normal tracer uptake as zero, mildly reduced uptake as one, moderately reduced uptake as two, severely reduced uptake as three, and absent uptake as four [14]. The rest score of each segment was subtracted from the corresponding stress score to calculate the difference score. If the rest score was higher than the stress score in a particular segment, the difference score was set to zero. By summing up the scores of all 17 segments, the summed rest score (SRS), summed stress score (SSS), and summed difference score (SDS) were calculated. Averaged scores from both readers were used for analysis.

Additionally, a visual analysis of the shifted and non-shifted reconstructions with regard to the presence or absence and extent (given as a percentage of the entire left-ventricular myocardium) of ischemia and/or scar was performed by two experienced readers in consensus.

Misalignment artifacts (MA) for rest and stress datasets were defined as the extent of perfusion defect that was unmasked by MRAC shifting. It was quantified by calculating the difference of the summed scores based on the non-shifted and shifted images for both stress (i.e., SSS_non-shifted_ − SSS_shifted_ = MA_stress_) and rest (i.e., SRS_non-shifted_ − SRS_shifted_ = MA_rest_).

### Myocardial creep

**Fig. 2:**
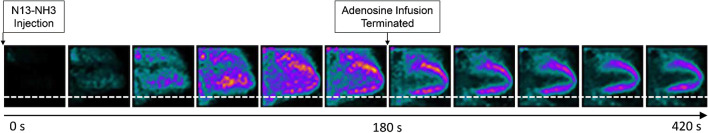
Myocardial creep. Selected frames oriented as vertical long axis during adenosine-induced stress 13N-NH3 PET MPI. Note the cranial dislocation of the heart after termination of the adenosine infusion

Myocardial creep was defined as the gradual caudo-cranial (i.e., along the *z*-axis) movement of the diaphragm, and subsequently the heart, after termination of the adenosine infusion (Fig. 2), and was quantified as the difference in millimeters along the *z*-axis between the most cranial point of the diaphragm in the stress and rest MRAC images, respectively.

A myocardial creep-induced misalignment artifact (MCMA) was defined as the extent of reversible perfusion defect that was unmasked by MRAC shifting. It was assessed by calculating the difference of the SDS based on the non-shifted and shifted images (i.e., SDS_non-shifted_ − SDS_shifted_ = MCMA).

### Statistical analysis

Continuous variables are expressed as mean ± standard deviation (SD) or as median with interquartile range (IQR), if not normally distributed. Categorical variables are presented as frequencies or percentages. The Kolmogorov-Smirnov test was used to test for normal distribution. *p* values for paired variables were calculated by paired *t* test if they were normally distributed and by Wilcoxon’s signed-rank test if the data was non-parametric. A one sampled *t* test was used to determine if mean MCMA differed from zero. For correlation analysis of non-parametric samples, the method of spearman (Spearman’s rho) was used. A two-way mixed consistency intraclass correlation coefficient was calculated for assessing inter-rater reliability. Pearson’s chi-square test was used to assess for statistically significant differences between categorical variables. A *p* value < 0.05 was considered statistically significant. SPSS version 25 (IBM Corporation, Armonk, NY, USA) was used for statistical analysis.

## Results

### Study population

Baseline characteristics are given in Table 1.

**Table 1:** Baseline characteristics (*n*=28)

Demographics	
Age (years)	36 ± 16
Male	24 (86%)
Female	4 (14%)
BMI (kg/m^2^)	26 ± 3.3
Risk factors	
Hypertension	4 (14%)
Dyslipidemia	2 (7%)
Diabetes	0 (0%)
Positive family history	7 (25%)
Active or past smoking	12 (43%)
Current cardiac symptoms	
None	24 (86%)
Atypical angina	2 (7%)
Non-anginal chest pain	2 (7%)
Current medication	
Betablocker	1 (4%)
ACEI or ARB	5 (18%)
Diuretics	1 (4%)

Values provided are mean ± standard deviation or absolute numbers and percentages (in brackets)

*BMI* Body mass index, *ACEI* angiotensin-converting enzyme inhibitor, *ARB* angiotensin receptor blocker

### MRAC shifting

A manual MRAC shift was deemed necessary in 25 (89.3%) stress datasets and 12 (42.9%) rest datasets. The median cardiac misalignment was 14 mm (IQR = 11–18) for the stress and 0 mm (IQR = 0–8) for the rest datasets (*p* < 0.001).

### PET images

In an analysis confined to datasets for which an MRAC shift was deemed necessary (*n* = 37), median SSS and SRS in non-shifted images were 8 (IQR = 5–14) and in shifted images 3 (IQR 1–5). Non-shifted values were shown to be significantly higher (*p* < 0.001). MA_stress_ and MA_rest_ correlated significantly with the cardiac misalignment (*r* = 0.58, *p* < 0.001). Median MA_stress_ was 7 (IQR = 3–10), and median MA_rest_ was 3 (IQR = 2–3). Median MCMA was 5 (IQR = 2–8).

The comparison of shifted versus non-shifted SSS, SRS, and SDS in all datasets where an MRAC shift was performed is presented in Table 2. Of note, all scores were found to be significantly lower after shifting.

**Table 2:** Comparison of non-shifted and shifted PET datasets - semiquantitative analysis

	*n*	Non-shifted	Shifted	*p* value
SSS	25	12 (7–15)	4 (2–6)	0.005
SRS	12	5 (4–7)	2 (1–4)	0.003
SDS	25	8 (2–11)	1 (0–2)	< 0.001

Values provided are median and interquartile ranges (in brackets)

*SSS* summed stress score, *SRS* summed rest score, *SDS* summed difference score

The results of the visual diagnosis towards ischemia and/or scar are provided in Table 3. Apparent areas of ischemia in the non-shifted images were located mostly in the anterolateral (*n* = 18, 64%), anterior (*n* = 13, 46%), and inferolateral (*n* = 12, 43%) left-ventricular myocardium. By contrast, the anteroseptal (*n* = 2, 7%), inferoseptal (*n* = 2, 7%), and the inferior (*n* = 1, 4%) as well as the apical region (*n* = 2, 7%) were less affected.

**Table 3:** Comparison of non-shifted and shifted PET datasets - visual diagnosis

		Non-shifted	Shifted	*p* value
Ischemia		20 (71%)	0 (0%)	< 0.001
Extent of ischemia	< 5%	2 (7%)	0 (0%)	< 0.001
	5–9.9%	4 (14%)	0 (0%)	
	10–20%	9 (32%)	0 (0%)	
	> 20%	5 (18%)	0 (0%)	
Scar		20 (71%)	18 (64%)	0.567
Extent of scar	< 5%	5 (18%)	5 (18%)	0.563
	5–9.9%	8 (29%)	10 (36%)	
	10–20%	7 (25%)	3 (11%)	
	> 20%	0 (0%)	0 (0%)	

Values provided are absolute numbers and percentages (in brackets). This analysis includes all datasets. If no shift was deemed necessary, the non-shifted images were used

### Myocardial creep

Median myocardial creep was 8 mm (IQR = 0–18). MCMA correlated moderately with myocardial creep (*r* = 0.541, *p* = 0.005). By contrast, neither MA_stress_ nor MA_rest_ correlated with myocardial creep.

### Inter-reader agreement

Inter-reader agreement was excellent with an intraclass correlation coefficient of 0.94 (95% CI 0.9–0.97) and 0.95 (95% CI 0.92–0.97) for SSS and SRS, respectively (both *p* < 0.001).

## Discussion

**Fig. 3:**
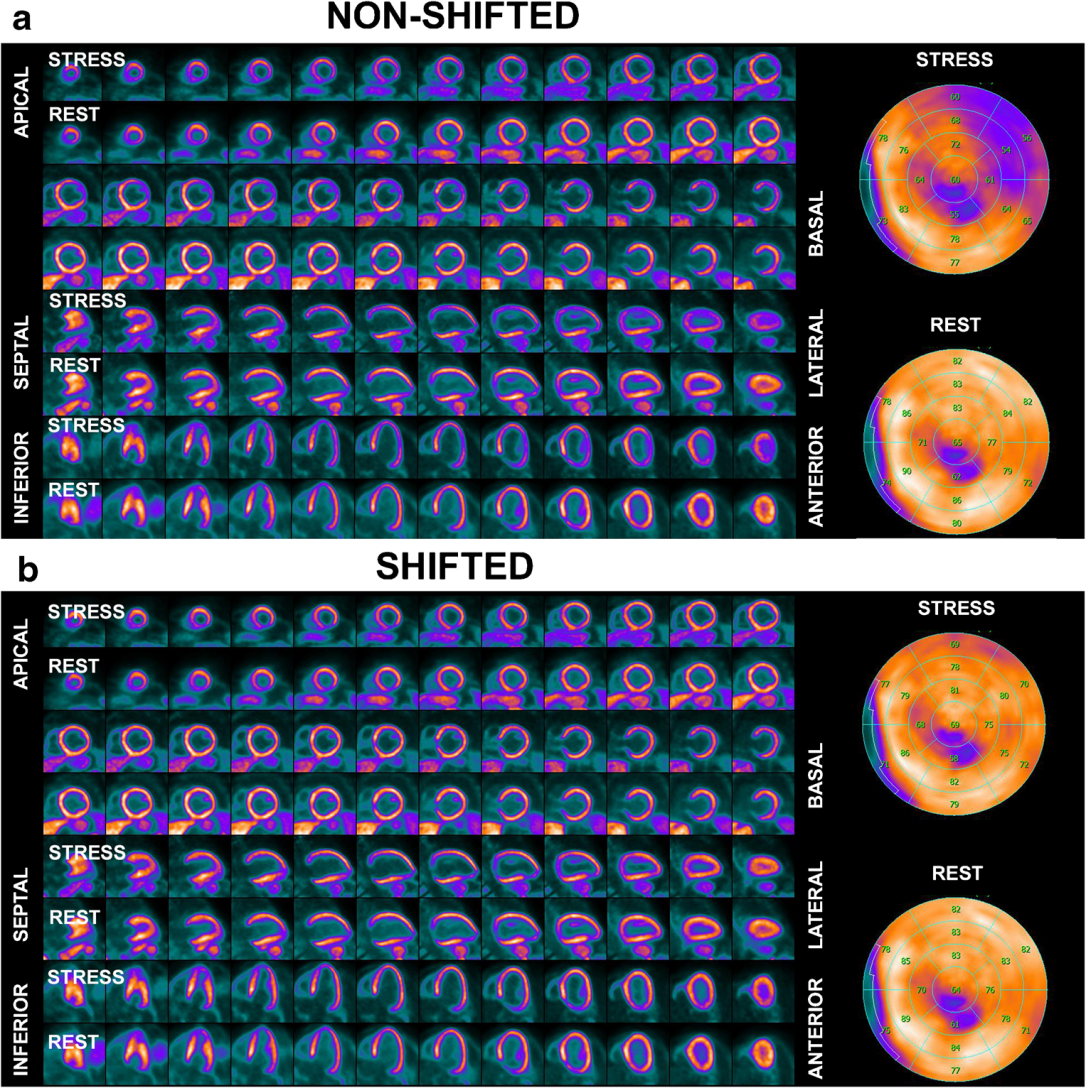
Example of a myocardial creep-induced misalignment artifact. **a** Non-shifted images depict a reduction in anterolateral counts in the stress images, mimicking a reversible perfusion defect. **b** Manual MRAC shifting leads to near-complete normalization in the anterolateral wall, as shown in the shifted images, unmasking the apparent perfusion defect as an artifact

In the present study, we found that misalignment between MRAC maps and PET datasets is a common finding during PET/MR MPI using adenosine stress and is causing misalignment artifacts in a substantial proportion of patients. Hence, careful examination and, where necessary, manual adjustment of the co-registration are mandatory. In the present study, manual MRAC shifting led to a substantial reduction in apparent perfusion defects as compared with non-shifted images. In fact, semi-quantitative scores indicating perfusion abnormalities were substantially lower after MRAC shifting, and visual analysis revealed that the latter led to complete normalization in 30% (8/26) of patients in whom perfusion abnormalities were present according to non-shifted images. In the vast majority of patients, apparent perfusion defects unmasked as potential artifacts by MRAC shifting affected the anterolateral, inferolateral, and anterior left-ventricular myocardium. Furthermore, we found that cardiac misalignment occurs more frequently and more extensively during adenosine-induced stress than during resting conditions (Fig. 3).

Additionally, there was a moderate but significant correlation between the extent of artificial perfusion defects and cardiac misalignment. Hence, it may be hypothesized that myocardial creep may be a substantial cause of this artifact.

Previous studies have demonstrated that vasodilator stress agents may inflict a change in breathing levels [6, 11]. In our study, we found that the presence and extent of reversible perfusion defects, which were unmasked by MRAC shifting, interrelate with the adenosine-dependent myocardial creep. Vasodilator stress causes a temporary change of breathing levels, leading to a change of the position of the diaphragm and, consequently, of the heart (Fig. 1B). Termination of the vasodilator is then followed by gradual normalization of the breathing level, paralleled by a slow cranial movement of the diaphragm and subsequently of the heart, i.e., myocardial creep. The chronology of these mechanisms is of importance, because MRAC, as well as dynamic and static PET datasets, is acquired at different points in time along a PET/MR MPI protocol and, hence, at different breathing levels and, consequently, with varying positions of heart. This causes a misalignment of MRAC for gated and static PET datasets if the MRAC scans are acquired during adenosine infusion and vice versa, and a misalignment of MRAC for the first frames of the dynamic PET dataset if acquired prior or after adenosine infusion.

MCMA can be considered as a sub-entity of MRAC artifacts along with the misalignment artifacts in rest datasets, which are well known from PET/CT and which may be caused by subtle patient motion. The latter occurs less frequently and affects images less profoundly than MCMA. In fact, in the present study, we found that 71% of the studies potentially would have been false-positively reported as showing ischemia if no review and manual MRAC shifting had been performed.

Recently, Lassen et al. [5] have described the occurrence of rest misalignment artifacts during PET/MR. In their study, they observed a cardiac misalignment in 55% of the rest 13N-ammonia scans, which is comparable to the rate of misalignment under resting conditions (42.9%) in the present study. While Lassen et al. reported severe underestimation of myocardial uptake in case of misalignment of > 10 mm, we found a correlation between cardiac misalignment and misalignment artifact size, which suggests a more continuous rather than a dichotomous relationship between misalignment and artifact.

To avoid false-positive findings in PET/MR MPI, we recommend a thorough and systematic review of MRAC maps and PET datasets to identify and, if possible, correct for any spatial misalignment. However, it is important to mention that the corrective shifting method proposed in our study represents a suboptimal solution to the problem, because it constitutes a rigid rather than a non-rigid correction of co-registration. Furthermore, AC is not confined to local correction only, and the shifting procedure per se may cause other structures to move in a wrong spatial position relative to the heart, potentially introducing other more subtle artifacts. It was beyond the aim of the present study to develop and validate a highly complex, proprietary, and likely vendor-dependent correction algorithm. The current study emphasizes the importance and frequency of cardiac misalignment during PET/MR and extends our understanding of potential mechanisms causing the related artifacts. Therefore, our results may rather set the ground for future alternative and preferably simple-to-implement solutions. One such alternative approach could lie in the temporal relocation of the stress MRAC scan. If the MRAC scan were not performed at the beginning of the PET acquisition but rather at the end, the effect of vasodilator-induced myocardial creep could potentially be diminished, and the breathing level during MRAC acquisition would expectedly be the same as during the gated and static PET acquisition. However, with such a procedure, the dynamic PET acquisition and particularly the first frames would undeniably and frequently be affected by misalignment. This would again require careful review and correction especially because the first frames comprise the data on the tracer inflow. Correction of dynamic PET datasets, however, may be much more challenging to achieve, as it would require frame-by-frame review and adjustments. The acquisition of two separate MRAC maps for the dynamic, as well as the static, PET datasets (i.e., acquisition of Dixon sequences before dynamic PET and additional Dixon sequence acquisition after static PET) could represent an elegant solution, potentially resulting in a lower rate of misalignment artifacts and should be incorporated by vendors. Nevertheless, a careful review of co-registration would remain crucially important because other factors than myocardial creep may lead to misalignment, as implied by the relatively high reported rate of misalignment during rest acquisition.

A different approach to overcome the problem of misalignment artifacts could potentially lie in motion correction. Such algorithms are in principle based on the cardiac and respiratory binning of PET data acquired in list mode, derivation of motion fields, and finally reconstruction of cardiac and respiratory motion-corrected images. Several small pilot studies have hinted at the potential of motion correction for cardiac 18F-FDG PET/MR by demonstrating improved PET image quality with less motion-induced blurring after correction [15, 16]. However, while correction algorithms may account for respiratory and cardiac motion, it remains to be elucidated by future studies whether these techniques could also be of value in addressing the issue of misalignment between PET data and MRAC maps in a setting of PET MPI where upward creep occurs after vasodilator-induced stress.

### Limitations

It may be perceived as a limitation that we did not use a similar definition of myocardial creep as proposed by Koenders et al. [8]. We considered the change of breathing and myocardial creep and its induced artifacts as a continuous and not dichotomous variable. Hence, we felt that recording the diaphragmatic displacement is a more accurate parameter to assess the severity of this phenomenon. Nevertheless, in order to stay in line with the methodology of Koenders et al. [8] and Friedman et al. [9], we defined myocardial creep as a movement along the caudo-cranial axis. However, as cardiac misalignment is most likely not entirely confined to motion within one dimension only, the MRAC shift was executed in all three axes in the present study. This discrepancy may have led to an underestimation of the correlation between MCMA and myocardial creep. Further detailed research on myocardial creep is warranted to understand the three-dimensional aspect of myocardial creep and to provide an all-encompassing definition.

Furthermore, due to the lack of a reference standard, we cannot comment on the validity of the normalization of perfusion caused by the shifting procedure. Additionally, we cannot comment on the potential impact of other factors such as whole-body or breathing movement as no motion detection and/or correction algorithms were applied during PET/MR image acquisition. However, since adenosine was the only changing variable between rest and stress image acquisition, it is reasonable to assume that myocardial creep may be the most substantial cause of the reported misalignment artifacts.

Finally, this was a single-center, single-vendor study. Any extrapolation of our results to scanners of other vendors may only be done with caution.

## Conclusion

Cardiac misalignment of MRAC maps and PET datasets commonly occurs during adenosine stress MPI on a hybrid PET/MR device and may lead to an increase in potentially false-positive findings. Our results suggest that myocardial creep may be a substantial cause of this. A careful, systematic review focused on the spatial misalignment between MRAC and PET images and its eventual correction through MRAC shifting is mandatory.

## Data Availability

Participants of this study did not agree for their data to be shared publicly, so supporting data is not available.
